# Neutralizing antibodies to Severe Fever with Thrombocytopenia Syndrome Virus in general population, Shandong Province, China

**DOI:** 10.1038/s41598-018-33884-z

**Published:** 2018-10-18

**Authors:** Dexin Li, Lijun Shao, Yu Bi, Guoyu Niu

**Affiliations:** 1 School of Public Health and management, WeiFang Medical University, Weifang, 261053 China; 2 Laboratory Institute for Viral Disease Control and Prevention, China CDC, 155 Chang Bai Road, Chang Ping District, Beijing, 102206 China

## Abstract

Severe fever with thrombocytopenia syndrome (SFTS) is an emerging infectious disease caused by SFTS virus (SFTSV) in East Asia. The research on seroprevalence of SFTSV in healthy people and risk factors had been detailed. However, the levels of neutralizing antibodies against SFTSV in general population were currently unclear. In the present study, we tested 1375 healthy persons from Penglai County, eastern China, for SFTSV neutralizing antibodies; 0.58% (8/1,375) was positive and the positive rates were not significantly different among people at different age groups, occupations and genders. Besides, a follow-up study was conducted and the titer of neutralizing antibodies decreased over time in all eight people but one, and the neutralizing antibodies of five lasted for the entire study period of seven years. Our results suggesting that subclinical infection or a relatively mild form of SFTS illness is occurring in this population, but a small percentage of sera have neutralizing capacity to SFTSV. Hence, most people are just susceptible to SFTSV infection.

## Introduction

Severe fever with thrombocytopenia syndrome (SFTS) is an emerging hemorrhagic fever in East Asia that was caused by SFTS virus (SFTSV), a novel phlebovirus in Bunyaviridae family^1^. SFTS was first reported during 2010 in China, where it was most prevalent in rural area of Henan, Hubei and Shandong provinces and later, had been reported in Korea and Japan^2,3^. The major clinical symptoms and laboratory abnormalities of SFTS are fever, thrombocytopenia, leukopenia, bleeding tendency and SFTS patients usually die due to multiple organ failure^1^. No effective specific treatment for SFTSV infection other than supportive care had been established. SFTSV is transmitted by tick bite because the virus was detected from Haemaphysalis longicornis ticks at every stage of development^1,4^. Occasionally, the disease could also be transmitted from person to person through contact with infected blood or mucus^5,6^. Some animals are considered to be host of SFTSV including domestic animals such as goats, cattle, dogs, chickens, pigs and small mammals such as shrews and rodent^7–9^.

Although most human SFTS cases were sporadic, the disease had obvious epidemiological characteristics. Geographically, it had been shown that cases of infection with SFTSV were found predominantly in hilly rural areas^10^. The patients were mostly laborers working in the field or rearing livestock. In term of time, most SFTS cases were reported between the months of May–July in China^11^. This was consistent with the seasonal abundance of ticks^12^. Various epidemiological studies had investigated the prevalence of SFTSV in general populations and recognized that age was the critical risk factor or determinant for SFTSV morbidity and mortality^13^.

The research on seroprevalence of SFTSV in healthy people and risk factors had been detailed, however, reports associated with neutralizing antibodies to SFTSV in general population are rare. According to the information system for disease control and prevention, 180 cases of SFTS and 35 deaths were reported in Penglai County, Shandong Province, China, from 2010 to 2017. To investigate the characteristics of neutralizing antibodies in general population, a serological investigation was conducted in Penglai area in 2011 and follow-up surveys were conducted in 2014 and 2017 respectively. In our study, ELISA method was adopted as a screening and neutralization assay was used to confirm the presence of neutralizing antibodies against SFTSV in serum samples of people of different age groups, occupations and genders.

## Results

A total of 1,375 healthy persons were enrolled in our study with each group 150 to 200 persons. Of which, 44.15% (607/1,375) were male and 55.85% (768/1375) were female while 21.02% (289/1375) were students and 78.98 (1086/1375) were farmers engaged in agriculture activities.

### Seroprevalence of SFTSV infection tested by serological test

Overall, 3.85% (53/1,375) of general population were serum antibody positive to SFTSV by ELISA (Table 1). And all positive samples were confirmed by immunofluorescence assay (IFA). The SFTSV antibody positive persons were distributed in all age groups. Seropositive rate was higher in age groups 40 ~, 50 ~and 60 ~than that in other groups, and it was significantly different among the age groups (χ^2^ = 17.736, P < 0.05). 4.45% (27/607) male were serum antibody positive to SFTSV and 3.39% (26/768) female were serum antibody positive to SFTSV. The difference of seropositve rate was not statistically significant between male and female (χ^2^ = 1.033, P > 0.05). Seropositive rate was significant different between students (0.69%, 2/289) and farmers engaged in agriculture activities (4.7%, 51/1086) (χ^2^ = 9.875, P < 0.05) (Table 1).

**Table 1:** Characteristics of total antibodies and neutralizing antibodies to severe fever with thrombocytopenia syndrome virus in general population, Shandong Province, China.

Characteristics	No. participants	Antibody-positive participants				Neutralizing antibody-positive participants			
		No. (%)	95%CI	P value	χ^2^	No. (%)	95%CI	P value	χ^2^
Sex				0.309	1.033			0.461	0.543
M	607	27 (4.45)	2.80–6.10			2 (0.33)	0.12–0.78		
F	768	26 (3.39)	2.11–4.67			6 (0.78)	0.16–1.40		
Occupation				0.002	9.875			0.875	0.025
Farmers	1086	51 (4.7)	3.44–5.96			7 (0.68)	0.20–1.16		
Students	289	2 (0.69)	0.26–1.64			1 (0.68)	0.33–1.63		
Age, years				0.013	17.736			0.997^§^	
0-	146	1 (0.68)	0.65–2.01			0 (0)	0.00–1.28		
10-	152	1 (0.66)	0.63–1.95			1 (0.66)	0.62–1.94		
20-	151	5 (3.31)	0.46–4.16			1 (0.66)	0.63–1.95		
30-	147	5 (3.4)	1.47–5.33			1 (0.68)	0.65–2.01		
40-	193	13 (6.74)	4.20–8.28			1 (0.52)	0.49–1.53		
50-	208	13 (6.25)	4.96–9.54			1 (0.48)	0.46–1.42		
60-	214	11 (5.14)	3.18–7.10			2 (0.93)	0.35–2.22		
70-	164	4 (2.44)	1.80–4.80			1 (0.61)	0.58–1.80		
Total	1375	53 (3.85)	2.83–4.87			8 (0.58)	0.18–0.98		

^§^Fisher exact test was used to compare groups.

### The positive rate of neutralizing antibody to SFTSV tested by neutralization assay

In our study, 0.58% (8/1,375) of the healthy persons was serum neutralizing antibody positive to SFTSV by neutralizing assay (Table 1). The neutralizing antibody positive persons were distributed in all age groups, but few in number. After combining two neighbor groups together, no significant difference among the age groups was found (p = 0.997, fisher exact test). Besides, the difference of neutralizing antibody positive rates was not statistically significant between male and female (χ^2^ = 0.461, P > 0.05), students and farmers engaged in agriculture activities (χ^2^ = 0.875, P > 0.05) (Table 1).

### The titers of neutralizing antibodies in sera

Neutralization test showed that 15.1% (8/53) of the sera with SFTSV antibodies detected by ELISA had the ability to neutralize virus, of which, the neutralization titers of four serum samples were all 1:10, and the rest were 1:40 (n = 1), 1:80 (n = 2) and 1:640 (n = 1). Subsequently we conducted a follow-up survey to the eight persons according to registered information. We collected their blood samples for 2 times in 2014 and 2017 respectively, and seven of them agreed and one refused to donate blood samples for the second time. At last, 15 specimens were obtained and tested by neutralization assay. Results showed that the titer of neutralizing antibodies decreased over time in all but one person (No. 4), who had a higher neutralizing antibody titer in 2017 than in 2011, and the neutralizing antibodies of five lasted for the entire study period of seven years (Table 2).

**Table 2:** The titer of neutralizing antibodies of 8 people in follow-up study.

No. People	Gender	Age	Occupation	2011		2014		2017	
				Titer by neutralizing assay	Titer by ELIA	Titer by neutralizing assay	Titer by ELIA	Titer by neutralizing assay	Titer by ELIA
1	F	40	farmers	10	800	0	400	0	200
2	F	10	students	10	400	0	100	NT	NT
3	F	35	farmers	10	800	0	200	0	100
4	M	51	farmers	10	1600	40	3200	40	1600
5	M	22	farmers	80	3200	40	800	40	800
6	F	42	farmers	80	3200	40	1600	20	800
7	F	59	farmers	640	6400	320	6400	160	3200
8	F	38	farmers	40	1600	40	1600	20	400

NT, no test performed because serum was not available.

## Discussion

In our study, the positive rate of SFTSV antibody was found to be 3.85% in the general population. There were significant differences in positive rate among different age groups, and different occupations indicating that people at older age groups and farmers engaged in agriculture activities were susceptible to SFTSV infection. These results suggested that age and occupation were potential risk factors of SFTSV infection in general population of Penglai County. The positive rate in this study was similar to the reported percentage (3.3%) recorded in Shandong Province^14^. However, it was much higher than that reported in Jiangsu (0.44%), lower than that in Shanxi (4.7%)^15,16^. The discrepancy may be attributed to the season of collection, population size, geographic climatic factors and different methods. But it was worth noting that ELISA method was utilized in all of these studies. Considering that the effects of non specific reactions caused by ELISA method were not excluded, we used the ELISA method as a screening experiment and the positive samples were confirmed by IFA.

Results showed that 0.58% (8/1,375) of sera were positive for SFTSV neutralizing antibodies, which was far lower than the result of ELISA (3.85%, 53/1375). This imbalance may be due to that a variety of antibodies including neutralizing antibodies were produced in virus infection but the durations of them in the body were different. These results suggested that most sera (84.9%, 45/53) positive for SFTSV had no protective effect and the majority of population in this region was still in the threat of SFTSV infection. As the neutralization assay was recognized as the gold standard for detecting virus antibodies, we believed that the positive rate of 0.58% represented the lowest positive rate of SFTSV infection in the detected population.

The result of neutralizing assay suggested that the natural SFTSV infection did exist in Penglai region but its prevalence was low. Through statistical analysis, we know that the positive rate of neutralizing antibodies is not statistically significant among different ages, occupations and genders. In the present study, eight people with neutralizing antibodies in the sera denied the experience of hospitalization for illness resembling SFTS in the past five years, indicating that SFTSV cause subclinical infections or a relatively mild form of SFTS illness in human. Consequently, the number of actual infections was more than the number of hospitalized patients but the proportion between them still needed further study.

Recent studies have shown that 100% of SFTS confirmed cases produce neutralizing antibodies to SFTSV and can last for four years with a decrease in titers^17^. We also performed a follow-up study of eight people with neutralizing antibodies in 2014 and 2017. By comparing the results of neutralizing antibodies in three surveys (2011, 2014 and 2017), we found that the titers of neutralizing antibodies in general population were low except for No. 7 (640) and none of them showed obvious clinical symptoms. That probably because mild strain of SFTSV caused low immune responses in the body or the neutralizing antibodies decreased fast over time. In additional, the neutralizing antibodies of five people lasted for seven years, while the rest of others vanished within four years. That suggested the titer of neutralizing antibody was positively correlated with duration of protection. As to be expected, the titer of neutralizing antibody decreased over time in all but No. 4; this increase may have caused by SFTSV reinfection. However, it was reported that the seroprevalence rate of SFTSV in healthy population was <1% and the chance of reinfection of a person with SFTSV was low^12^. We can not exclude that No. 4 people could have been infected with other similar phleboviruses. Few data about neutralizing antibody in healthy population are available for comparison. Consequently, more studies are needed.

In summary, our findings suggest that subclinical SFTSV infections or a relatively mild form of SFTS illness affects humans in Penglai County. We confirm that a large percentage of healthy people did not have the ability to neutralize the virus and they may be infected by SFTSV. A few of people with mild SFTS illness could produce long-lasting neutralizing antibodies to SFTSV and its titers decreased over time. We do not know the characteristics of the neutralizing antibodies against SFTSV, which need to be further investigated.

## Methods

### Ethics approval

The study was reviewed and approved by the Ethic Committee of China Center for Disease Control and Prevention(China CDC). All study objects had signed an informed consent document prior to participation. A signed informed consent was required from their parents or legal guardian for minors. All data analyzed were anonymized. Human research was conducted in compliance with the Helsinki Declaration.

### Study site

**Figure 1:**
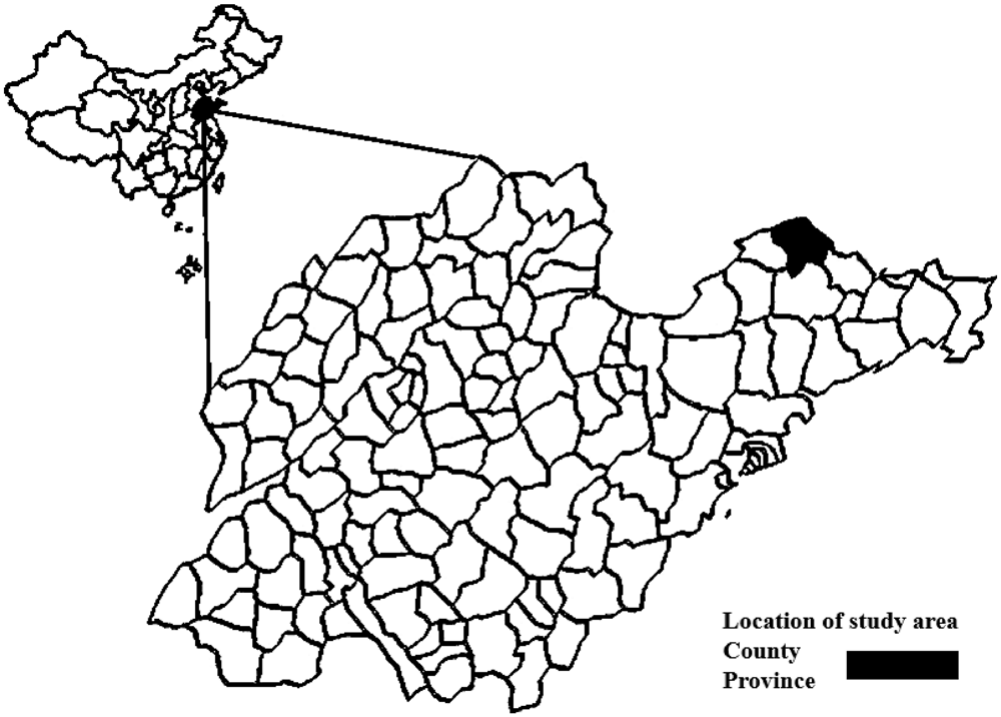
Location of Shandong Province in China (left) and the location of Laizhou country within the province where blood samples were collected.

Penglai County located in east longitude 120°34′ and 121°04′ and between north latitude 37°26′and 37°48′. It had a total area of 1, 128 square kilometers and a total population of ≈449,000, of whom 80% live in rural areas (Fig. 1).

### Serological investigation

Among the towns that had reported SFTS cases, we randomly selected 40 villages as target villages to collect serum specimens. In these selected villages, people were divided into 8 age groups as 0, 10, 20, 30, 40, 50, 60, and ≥70. In each group 150–200 persons were randomly selected to collect serum specimens in July, 2011. For the survey, the recruited persons were people who had never been hospitalized because of illness resembling SFTS in the past five years. 5 ml of blood was drawn from each person and shipped to the Penglai CDC on ice. Serum was separated by centrifugation and frozen at −80 °C until use. All tests of samples from serological investigation in 2011 were conduct in Laboratory Institute for Viral Disease Control and Prevention (Beijing), in 2012.

### Serological test

Sera were tested for antibodies to SFTSV by using a double-antigen sandwich ELISA kit (Xinlianxin Biomedical Technology Limited, Wuxi, China). Recombinant nucleoprotein (NP) of SFTSV was used as antigen for coating plates. The experiment included negative control, positive control and blank control. To each well 100 µl of a sample was added except for control wells, and then incubated at 37 °C for 30 minutes. After washing five times, each well was added 100 µl horse radish peroxidase (HRP) labeled reagent, then the plate was incubated at 37 °C for 30 minutes. After washing five times, a chromogenic agent A and B solution were added to each well to develop the color and the plate was read at 450 nm for optical density (OD). A serum sample was considered to contain SFTSV specific antibody when absorbance of the sample ≥ threshold value (cutoff). The threshold value = 0.10 + the average OD value of the negative control (if the OD value of a negative control was less than 0.04, it was considered as 0.04). In our study, undiluted samples were utilized to determine whether the samples were positive for antibodies against SFTSV. The ELISA results were confirmed by IFA. After the samples were diluted 1:10, 1:20, 1:40 and1:80 in phosphate-buffered saline (PBS)-Tween buffer, the IFA was performed and immunofluorescence was observed using an epifluorescence microscope. Goat anti-human IgG Fc-FITC (abcam) was used as second antibody. Positive and negative controls were also used. In our study, a titre of 1:40 was considered to be the most appropriate dilution.

### Neutralization Assay

A microneutralization assay was performed to detect neutralizing antibodies against SFTSV as described previously^8^. Briefly, samples were diluted in 2-fold increments from 1:5 to 1:640. Each dilution of serum samples was mixed with an equal volume of 100 median tissue-culture infectious doses of SFTSV (strain HB29) and incubated at 37 °C for 1.5 hours. The mixture was then added to a 96-well plate containing Vero cells in quadruplicate. The plates were incubated at 37 °C in a 5% carbon dioxide atmosphere for 7 days. Viral infection was detected via immunofluorescence assays with mouse polyclonal antibodies against SFTSV (AbMax) and rabbit anti-mouse IgG-FITC (abcam). The end-point titer was expressed as the reciprocal of the highest dilution of serum that prevented infection.

### Follow-up survey

According to the result of neutralization assay, we conducted a follow-up study of people with neutralizing antibodies against SFTSV. Blood samples were obtained two times during a seven-year period and were tested to determine the titers of neutralizing antibodies. The ELISA and micro-neutralization assay of samples from follow-up surveys were performed in WeiFang Medical University (Weifang), in 2017.

### Statistical analysis

Statistical analysis was performed using SPSS 21.0 software and P < 0.05 was considered as statistically significant difference. Chis-quare or Fisher’s exact test were used for categorical variables, where appropriate.

## Data Availability

The datasets generated during and/or analysed during the current study are available from the corresponding author on reasonable request. All data generated or analysed during this study are included in this published article.
